# They Brought the Hospital to Me

**DOI:** 10.5694/mja2.70281

**Published:** 2026-09-01

**Authors:** Hugh N. Burrill

**Affiliations:** ^1^ Faculty of Medicine Nursing and Health Sciences Australian and New Zealand Intensive Care Research Centre, School of Public Health and Preventive Medicine, Monash University Melbourne Victoria Australia

**Keywords:** cardiac arrhythmias, consumer health information, embolism and thrombosis, extracorporeal circulation, intensive care, paramedics, physician–patient relations, quality of life, retrieval medicine

## Abstract

I collapsed at the gym when a massive pulmonary embolism caused my heart to stop. Unbelievably I survived thanks to a clinical trial delivering an extracorporeal membrane oxygenation (ECMO) heart–lung bypass machine directly to me at the gym. Hopefully out‐of‐hospital ECMO becomes an accepted emergency strategy to save more lives.

One day in 2023, while exercising at my local gym, I suffered a cardiac arrest due to a massive pulmonary embolism.

A very large blood clot dislodged from veins in my right leg. It moved through my heart and got stuck in my lungs. This blockage made it impossible for my heart to pump, causing it to arrest. The attending first responders were unable to restart my heart and lungs, so I couldn't be transported to the hospital.

That day was the luckiest day of my life. One of Australia's leading critical care hospitals happened to have embarked on a pilot clinical research trial exploring the lifesaving potential of pre‐hospital deployment of extracorporeal membrane oxygenation (ECMO) during extracorporeal cardiopulmonary resuscitation (ECPR) for refractory out‐of‐hospital cardiac arrest (OHCA) [1]. This clinical trial involved bringing the heart–lung ECMO machine out of the hospital to the site of my cardiac arrest. This saved my life.

My luck started with my wife, a retired doctor, and my gym buddies, who immediately started CPR. My luck didn't stop there. I still needed to meet the inclusion criteria for the clinical trial including being between the ages of 18 and 65 (I was 65), having a witnessed arrest with an initial rhythm of pulseless electrical activity (PEA), the arrest being between 9 AM to 5 PM Monday to Thursday with immediate bystander CPR (I arrested at 9.36 PM on a Monday), being within a 45‐min drive to enable the specialist team to achieve rapid cannulation and ECMO establishment at the scene (I was a 20‐min drive away). There were lots of other make‐or‐break inclusion and exclusion criteria. Today I find this long list of criteria unsettling but the fact that I'm alive today means that I was incredibly lucky and managed to tick all the right boxes to be recruited into the clinical trial (Figure 1).

**FIGURE 1:**
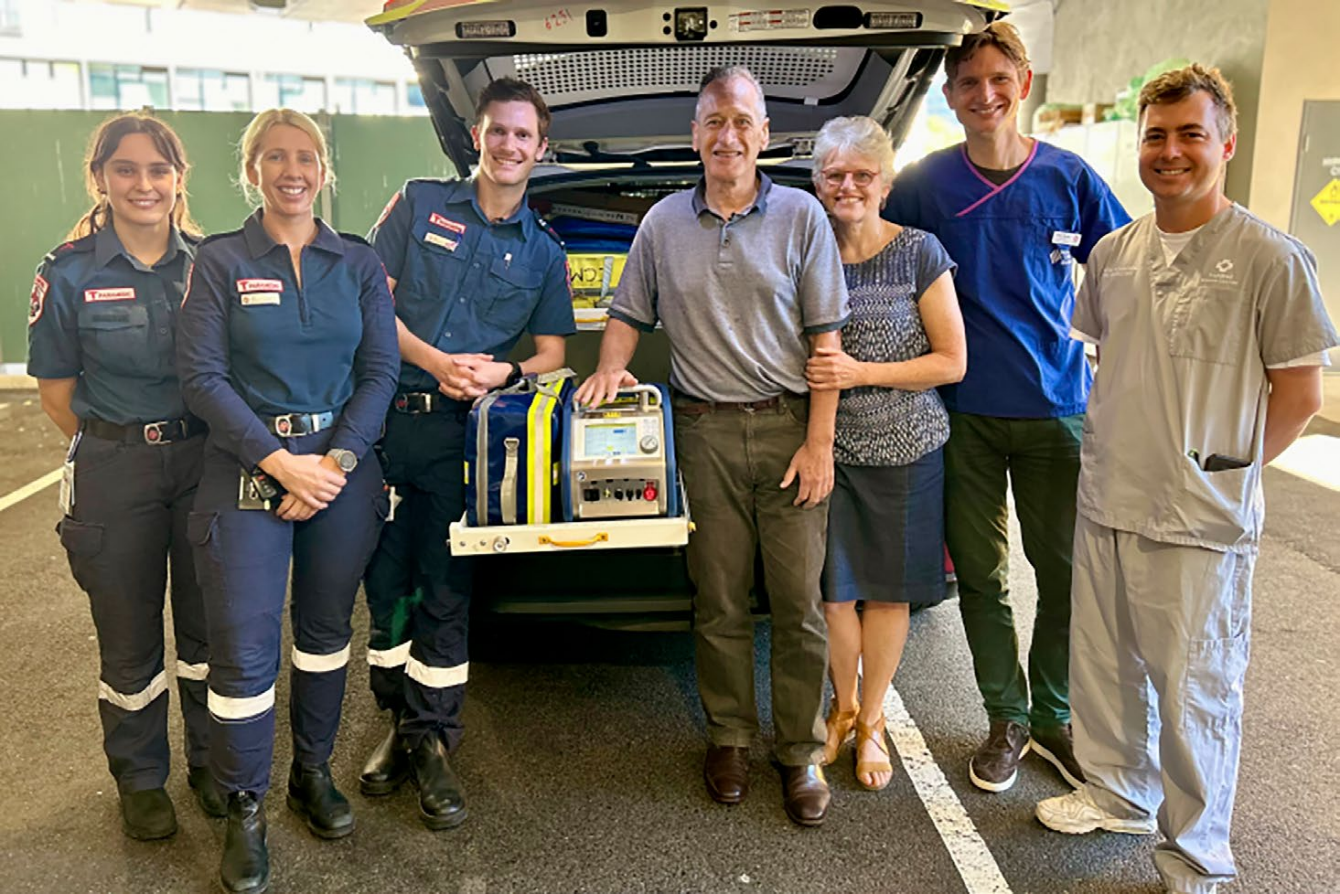
Pre‐hospital extracorporeal cardiopulmonary resuscitation (ECPR) team reunited with Hugh and his wife Meridee. Left to right: Ariadne Sakolevas, Jenna Schwarz, Alastair Miller, Hugh Burrill, Meridee Flower, Aidan Burrell and Sacha Richardson.

The probability of surviving this refractory PEA arrest without ECPR is reported to be less than 2% [2]. Being included in the trial likely improved my chances to about 40% [1]. This is still less than a 50/50 bet, but given the alternative, I'd happily take those odds!

After being stabilised on the ECMO equipment on the gym floor, I was transported to the hospital intensive care unit (ICU), fully sedated and maintained on ECMO until the clots in my lungs resolved enough for my heart and lungs to function independently. On Day 10, after ECMO had been removed and sedation withdrawn, I was transferred from ICU to the wards where I remained until I was discharged from hospital on Day 18. I moved onto a rehabilitation program, with my physical and psychological recovery addressed for the next few months.

My time in ICU was the most frightening of my life. I know that the care I received from the ICU and hospital staff was fantastic, but my mind was running in overdrive, and not in a good way. Once I began to come out of my sedated state, I was overwhelmed by hallucinations and in a constant state of paranoia. I feel incredibly embarrassed to admit that I was extremely fearful of the staff as I ‘heard’ them saying to each other that I was getting privileged treatment and didn't deserve this. I thought they were out to harm me. When anyone I didn't recognise came into the room, I fearfully asked them to stand back saying that I didn't know them. This also meant that I wasn't prepared to close my eyes, and after several days of this, I was extremely sleep deprived (not helped by the six broken ribs I had from the initial CPR performed at the gym). After eventually leaving hospital and regaining some degree of rational thinking, I felt the need to meet the ICU and hospital staff to apologise and let them know that it wasn't the true me that they saw, and that I truly appreciated the terrific care they took of me.

Once I was out of ICU and into the wards to continue my recovery, I was so incredibly blessed to have my wife constantly by my bedside (I still get teary out of gratitude when I think of this). Because my ability to read, write, comprehend and remember was significantly impaired, I was completely dependent on my wife to be my proxy on all matters both in the hospital and externally. Simple things like capturing the names of the medical staff that came and went, reading menus, managing outstanding matters at home that I was normally responsible for, corresponding with family and friends, and so many other basic human physical, cognitive and social functions. This made me realise how difficult and isolating it must be for someone in a similar situation without a family member or close friend to support them.

Later, when family and friends spoke to me about my cardiac arrest and my near‐death experience, they often asked about out‐of‐body awareness. My honest response is that if I did have such an awareness at the time, I don't remember it. I have almost no memory of the day of my arrest or the weeks afterwards, and of the very few images I do have—such as pressing my hand to my chest during the initial stages of the cardiac arrest—I find it impossible to determine whether they are real or created after the event.

The impact of my cardiac arrest has been profound and life changing. There have been negative residual effects due to a degree of hypoxic brain injury caused by the limited circulating oxygen before being placed on ECMO. I continue to have visual and cognitive impairments that impact my reading, writing, comprehension and memory, although these have partially improved overtime. There are physical changes. Numbness in the toes of my left foot, possibly due to the ECMO cannula. Like many ECPR patients, health challenges and resulting impairments have reduced my confidence to return to the workplace [3] and build on my previous executive career in global pharmaceutical research and development.

There have been positive impacts from the cardiac arrest. My family and I are obviously thrilled at the primary outcome, my survival. By surviving I was given a second chance to say all those things I could and should have said to loved ones but was yet to say. There is more. They have remarked on my greater level of emotional sensitivity and connectedness, and my increased appreciation of the importance of family and friends in my life.

Before my cardiac arrest, I was highly career focused and had a history of pushing myself in extreme sports. I spent years away from my family who stayed in Australia while I worked in Europe and the United States. I threw myself into racing in competitive endurance triathlons, runs and bike rides in Australia and overseas. Now, after my cardiac arrest, my exercise activities are more moderate and done for health and enjoyment rather than results. I am back at the gym each week with my wife and a group of close friends. On Saturdays we run in a community‐based 5 km ParkRun, and on Sunday I ride with a group of former work colleagues where the post‐ride coffee get‐together is more important than the ride itself.

Similarly, before the cardiac arrest my diet was one where I would eat and drink according to what I thought tasted good, without much thought to the health consequences. But I suspect that many people would have classified my diet as being relatively healthy. Since the arrest, I have switched to the diet my wife has been following for many years; a whole food, plant‐based diet. This, together with changing from a glass or two of red wine each night to not drinking any alcohol since the cardiac arrest 3 years ago, means I have transformed my blood pressure and cholesterol from levels requiring active management back to levels that my local doctor says are ‘perfect’.

As a person with lived experience, I now spend my time advocating for the needs of ECMO patients and their families in the wider community. This patient‐focused input guides clinical trials, including a study furthering the development of pre‐hospital ECPR. Research involvement grants me the pleasure of working with the same intensive care doctors who established me on ECMO while I was in cardiac arrest on the floor in the gym. An important part of future trials is aimed at validating the cost effectiveness of pre‐hospital ECPR, as the treatment is resource intensive [4].

This leads me to reflect on the innate human desire to save lives. We see this demonstrated when natural disasters threaten lives in a community, or when we put our own safety at risk to rescue a stranger, or when significant resources are deployed to rescue a wayward adventurer lost in the wilderness. As a result, I am optimistic that studies for OHCA, including ECMO, will be further developed and perhaps become an accepted emergency and intensive care strategy designed to save lives. Lives like mine.

## Author Contributions

**Hugh N. Burrill:** conceptualisation, investigation, validation, writing – original draft, writing – editing and reviewing.

## Funding

The author has nothing to report.

## Disclosure

Not commissioned; not externally peer reviewed.

## Conflicts of Interest

The author declares no conflicts of interest.

## Data Availability

There are no data in this manuscript.

## References

[1] S. A. C. Richardson , D. Anderson , A. J. C. Burrell , et al., “Pre‐Hospital ECPR in an Australian Metropolitan Setting: A Single‐Arm Feasibility Assessment – The CPR, Pre‐Hospital ECPR and Early Reperfusion (CHEER3) Study,” Scandinavian Journal of Trauma, Resuscitation and Emergency Medicine 31, no. 100 (2023): 1–8. 38093335 10.1186/s13049-023-01163-0 PMC10717258

[2] Z. Nehme , E. Andrew , S. Bernhard , and K. Smith , “Impact of Cardiopulmonary Resuscitation Duration on Survival From Paramedic Witnessed Out‐of‐Hospital Cardiac Arrests: An Observational Study,” Resuscitation 100 (2016): 25–31. 26774172 10.1016/j.resuscitation.2015.12.011

[3] C. L. Hodgson , A. M. Higgins , M. Bailey , et al., “Incidence of Death or Disability at 6 Months After Extracorporeal Membrane Oxygenation in Australia: A Prospective, Multicentre, Registry Embedded Cohort Study,” Lancet Respiratory Medicine 10, no. 11 (2022): 1038–1048. 36174613 10.1016/S2213-2600(22)00248-X

[4] M. Dennis , K. Shekar , A. J. C. Burrell , and National ECPR Working Group , “Extracorporeal Cardiopulmonary Resuscitation for Refractory Cardiac Arrest in Australia: A Narrative Review,” Medical Journal of Australia 220, no. 1 (2024): 46–53. 37872830 10.5694/mja2.52130

